# Cytosolic Glucose-6-Phosphate Dehydrogenase Is Involved in Seed Germination and Root Growth Under Salinity in *Arabidopsis*

**DOI:** 10.3389/fpls.2019.00182

**Published:** 2019-02-22

**Authors:** Lei Yang, Xiaomin Wang, Ning Chang, Wenbin Nan, Shengwang Wang, Mengjiao Ruan, Lili Sun, Sufang Li, Yurong Bi

**Affiliations:** ^1^Ministry of Education Key Laboratory of Cell Activities and Stress Adaptations, School of Life Sciences, Lanzhou University, Lanzhou, China; ^2^State Key Laboratory of Plateau Ecology and Agriculture, Qinghai University, Xining, China; ^3^Chongqing Key Laboratory of Molecular Biology of Plant Environmental Adaptations, College of Life Sciences, Chongqing Normal University, Chongqing, China

**Keywords:** germination, glucose-6-phosphate dehydrogenase, NaCl, NADPH oxidases, reactive oxygen species, root system architecture

## Abstract

Glucose-6-phosphate dehydrogenase (G6PDH or G6PD) is the key regulatory enzyme in the oxidative pentose phosphate pathway (OPPP). The cytosolic isoforms including G6PD5 and G6PD6 account for the major part of the G6PD total activity in plant cells. Here, we characterized the *Arabidopsis* single null mutant *g6pd5* and *g6pd6* and double mutant *g6pd5/6*. Compared to wild type, the mutant seeds showed a reduced germination rate and root elongation under salt stress. The seeds and seedlings lacking *G6PD5* and *G6PD6* accumulate more reactive oxygen species (ROS) than the wild type under salt stress. Cytosolic G6PD (cy-G6PD) affected the expression of NADPH oxidases and the G6PD enzymatic activities in the mutant *atrbohD/F*, in which the NADPH oxidases genes are disrupted by T-DNA insertion and generation of ROS is inhibited, were lower than that in the wild type. The NADPH level in mutants was decreased under salt stress. In addition, we found that G6PD5 and G6PD6 affected the activities and transcript levels of various antioxidant enzymes in response to salt stress, especially the ascorbate peroxidase and glutathione reductase. Exogenous application of ascorbate acid and glutathione rescued the seed and root phenotype of *g6pd5/6* under salt stress. Interestingly, the cytosolic G6PD negatively modulated the NaCl-blocked primary root growth under salt stress in the root meristem and elongation zone.

## Introduction

The oxidative pentose phosphate pathway (OPPP) is the major pathway of the production of NADPH, which is used for biosyntheses and redox balance in plant cells (Esposito et al., 2003; Kruger and von Schaewen, 2003; Hutchings et al., 2005; Cardi et al., 2011). The main regulatory step of OPPP is catalyzed by glucose-6-phosphate dehydrogenase (G6PDH or G6PD). The majority of NADPH in the cytoplasm is produced by G6PD and 6-phosphogluconate dehydrogenase (Huan et al., 2014). *Arabidopsis* genome-wide analysis indicates the presence of two cytosolic (cy-G6PD) and four plastidial (pla-G6PD) isoforms (Wakao and Benning, 2005). The cy-G6PD includes G6PD5 and G6PD6. Based on the difference in amino acid sequence, the pla-G6PD can be divided into P1, P2, and P0 type: P1 mainly exists in the chloroplast (G6PD1); P2 mainly exists in plastids and some non-oxygen cells (G6PD2, G6PD3), P0 is a non-functional enzyme (G6PD4) (Wakao and Benning, 2005). Many studies have indicated that G6PD plays an important role in plants to cope with stresses, including salinity and drought (Meyer et al., 2011; Liu et al., 2013; Huan et al., 2014; Wang et al., 2016). Certainly, salinity is a major environmental restriction for the growth of agricultural crops and negatively affects plant productivity (Hasegawa et al., 2000; Zhu, 2001; Dal Santo et al., 2012).

Salinity brings about water deficit and ion stress, which cause destabilization of cell membranes, inhibition of essential enzymes, overproduction of reactive oxygen species (ROS), and decrease in nutrient supply (Hasegawa et al., 2000; Dal Santo et al., 2012). ROS regulate many biological processes including seed germination and root growth in plants (Kwak et al., 2006; Dunand et al., 2007). It has been documented that ROS are produced through both enzymatic and non-enzymatic reactions in plants (Apel and Hirt, 2004; Ma et al., 2012). ROS directly originate from two ROS-generating NADPH oxidases, impairing stress inhibition of primary root elongation in *Arabidopsis* (Kwak et al., 2006; Jiao et al., 2013). However, continuously increased levels of ROS exceed cellular antioxidant capacity, thus are toxic to cells and affect all cellular biomolecules (Niforou et al., 2014; Jia et al., 2016). In *Arabidopsis* genome, there are 10 NADPH-oxidase catalytic subunit genes (*AtrbohA-J*) (Marino et al., 2012). NADPH oxidase controls shoot branching and reproductive organ development in tomato, and is required for pollen tube growth in tobacco (Sagi et al., 2004; Potocky et al., 2007). NADPH oxidases require NADPH to generate superoxide, which can be dismutated subsequently to hydrogen peroxide (Stampfl et al., 2016). In maize, ROS derived from NADPH oxidase is necessary for normal root growth (Liszkay et al., 2004). In bacteria, studies provide experimental evidence for a role of NADPH oxidase-derived ROS in establishing a relationship with pattern-triggered immunity in *Arabidopsis* (Stampfl et al., 2016). Such oxidative bursts are usually accompanied by transient oxidation of the cytosol (decreased NADPH levels) that triggers redox signaling and activation of the OPPP (Landi et al., 2016; Stampfl et al., 2016; Wang et al., 2016).

Plants can minimize the effects of salinity stress by removing excess ROS via increasing antioxidant enzyme activities (Yang et al., 2015; Landi et al., 2016). More recently, it is reported that G6PD plays a primary role during stress response by providing more NADPH for the antioxidant systems favoring ROS scavenging functions (Dal Santo et al., 2012; Landi et al., 2016). G6PD functions on modulating reduced glutathione levels in reed callus (Wang et al., 2008), establishing tolerance of red kidney bean roots to salt stress (Liu et al., 2007), and upregulating plasma membrane (PM) H^+^-ATPase activity, which results in the enhanced K^+^/Na^+^ ratio (Li et al., 2011).

In *Arabidopsis*, non-dormant seeds produce significant ROS during imbibition (Leymarie et al., 2012; Chen et al., 2014a). Seed germination and root growth are critical phases in the plant life cycle (Chen et al., 2014a; Wang et al., 2014). The ability of seeds to properly germinate depends on its oxidative status (Rajjou et al., 2012; Chen et al., 2014a). Over-accumulation of ROS causes oxidative damages to cellular components (Bailly et al., 2008; Parkhey et al., 2012). In plants, some hydrogen peroxide-scavenging substances protect seeds and roots from excessive oxidative damages, for example, ascorbate (Asc) and reduced glutathione (GSH) (Dal Santo et al., 2012; Chen et al., 2014a). GSH and Asc detoxify H_2_O_2_ mainly through the ascorbate-glutathione cycle, which is the most effective way to scavenge H_2_O_2_ in plants (Noctor and Foyer, 1998; Wang et al., 2016).

Based on the above studies, although the relationship between G6PDs and salt stress have been elucidated (Wang et al., 2008), function of G6PDs depends upon the developmental stage, organ/tissue, and species. In this work, we used the genetic and molecular approaches to study the function of cy-G6PDs. We characterized the function of *G6PD5* and *G6PD6*, which show enhanced tolerance to salt stress during seed germination and root growth, and functional interaction and synergism between G6PD and GSH during salt stress. We revealed a novel interplay between rbohD/F, ROS, ascorbate peroxidase (APX), and glutathione reductase (GR).

## Materials and Methods

### Plant Materials and Growth Conditions

*Arabidopsis* *thaliana* Col-0 was used as the WT plant. The T-DNA insertion mutants *g6pd5* (CS804669) and *g6pd6* (SALK_016157C) were purchased from the Arabidopsis Biological Resource Center^1^. The T-DNA in the *g6pd5* mutant is inserted in the coding region of At3g27300, and in the *g6pd6* mutant it is inserted in the coding region of *At5g40760*. The overexpression plants of *G6PD5* (*OE*#*1*, *OE*#*9*) and *G6PD6* (*OE*#*17*, *OE*#*21*) were generated by transforming the *G6PD5-* or *G6PD6-*containing constructs into WT. The double mutant *g6pd5/6* was generated by crossing *g6pd5* with *g6pd6*, followed by screening the F2 progeny for homozygosity at both loci by PCR genotyping. *atrbohD1* (CS9555), *atrbohF1* (CS9557), *atrbohD1/F1* (CS9558) were obtained from the Arabidopsis Biological Resource Center. Seeds were sterilized with 1.5% NaClO for 15 min, washed with sterile water for three times, placed at 4°C for 2–4 days and then planted on the half-strength Murashige and Skoog (½ MS) medium (pH 5.8) containing 1% sucrose and 0.8% agar at 23°C under 100–120 μmol photons ⋅ m^-2^ ⋅ s^-1^ with a 16 h/8 h light/dark photoperiod in the growth room.

### Phenotypic Analysis and Statistics

In all assays, WT, *g6pd5*, *g6pd6*, *g6pd5/6*, *OE*#*1*, *OE*#*9*, *OE*#*17*, and *OE*#*21* seeds (approximately 50 seeds for each replicate. For root elongation measurements, 15 seeds were used per replicate) were surface-sterilized. The seeds were sown on ½ MS medium with or without different concentration of NaCl and then incubated at 23°C with a 16 h/8 h light/dark photoperiod. The number of planted and germinated seeds was recorded 5 days after planting on the medium. Radicle emergence of >1 mm indicated seed germination. Three replicates were used for each treatment. Five-day-old seedlings with roots 1–1.5 cm long were transferred from agar plates containing ½ MS medium onto a new agar medium supplemented with different concentrations of NaCl. Increases in root length were measured after 3 days of treatment (Rosado et al., 2006; Nan et al., 2014). The length of the primary roots was measured with NIH Image software (Image J, version 1.43).

### Confocal Microscopy

Propidium iodide (PI) fluorescence was used to visualize the cells in root tips. Seedling roots were stained with PI (Molecular Probes, Sigma, United States) according to the method described by Mei et al. (2012). Roots were incubated with 10 μg/ml PI for 5–10 min at 23°C in the dark and then washed three times with ddH_2_O. The roots were then imaged under a confocal microscope (Olympus FV 1000; excitation 488 nm, emission 570–650 nm).

### Histochemical Staining and Assay of H_2_O_2_ Content

We used 2,7-dichlorodihydrofluorescein diacetate (H_2_DCF, Molecular Probes) to detect hydrogen peroxide (H_2_O_2_) accumulation in seeds and roots. Seeds of 12 h and roots of 5 days seedlings were treated with 20 μM H_2_DCF for 5 min, and fluorescence was monitored under a fluorescence microscope (Olympus FV 1000, excitation 488 nm and emission 500–550 nm).

For H_2_O_2_ content measurement, 10-day-old seedlings were soaked with in 150 mM NaCl solution for 12 h. Seedlings (0.3 g) were homogenized with 2 ml of 0.1% (w/v) TCA, then centrifuged at 10,000 ×*g* for 20 min at 4°C. The supernatant (0.5 ml) was mixed with 1 ml of 1 M potassium iodide for 1 h in the presence of 0.5 ml of 0.1 M Tris-HCl (pH 7.6). The absorbance was read at 390 nm and H_2_O_2_ content was determined using a standard curve.

### Antioxidant Enzyme and Activities of G6PD Assays

Ten-day-old seedlings were soaked in 150 mM NaCl solution for 12 h. After treatment, the enzymes extraction and activity determination of G6PD and antioxidant enzymes were carried out according to the method of Liu et al. (2007) and Wang et al. (2008). Briefly, crude enzymes were extracted in extraction buffer containing 50 mM Hepes-Tris (pH 7.8), 1 mM EDTA, and 3 mM MgCl_2_. The homogenate was then centrifuged at 12,000 ×*g* for 20 min at 4°C. The supernatant was used to determine enzyme activity.

For ascorbate peroxidase (APX) activity, the reagent was composed of 0.1 mM EDTA-Na_2_ and 0.3 mM ascorbate. The enzyme extract (100 μl) and 1 ml reagent were mixed in cuvette in the presence of 20 μl of 9 mM H_2_O_2_. The absorbance at 290 nm was recorded for 1min.

For catalase (CAT) activity, the experiment group contained 1 ml of 15 mM H_2_O_2_ and 100 μl of enzyme extract. The change of absorbance at 240 nm was recorded.

For glutathione reductase (GR) activity, 0.52 mM Tris–HCl (pH 7.5), 6 μM EDTA, 2 mM GSSG, 4 mM NADPHNa_4_, and crude enzyme (100 μl) were mixed into 3 ml. GR activity was measured at 340 nm for the initial 3 min of the reaction at 25°C.

For peroxidase (POD) activity, the enzyme extract (25 μl) was mixed with 1 ml of 20 mM guaiacol in the presence of 20 μl of H_2_O_2_ for 3 min. The change of absorbance at 470 nm was record.

Superoxide dismutase (SOD) activity was measured in test tube. Reaction solution contained 2 ml of 39 mM methionine solution, 2 ml of 0.225 mM nitroblue tetrazolium, 1 ml of 0.6 mM EDTA-Na_2_ and 1 ml of 0.012 mM riboflavin. One tube was incubated in the light for 30 min, and the other tube was incubated in dark for 30 min. After 30 min the absorbance at 560 nm was recorded using a spectrophotometer.

For G6PD activity assay, the reagent was composed of 50 mM Hepes-Tris (pH 7.8), 1 mM EDTA, and 3 mM MgCl_2_. The G6PD activity was analyzed by detecting NADPH formation at 340 nm in the presence of 0.5 mM D-glucose-6-phosphate disodium salt (Sigma) and 0.5 mM NADPNa_2_. To distinguish the activity of cytosolic G6PD isoforms, 1,4-dithiothreitol (DTT) was added into the reaction mixture.

### Determination of Glutathione Content, NADPH and NADP^+^ Content

Ten-day-old seedlings were soaked in 150 mM NaCl solution for 12 h. After treatment, the glutathione content was measured using the GSH content determination kit (Cat# BC1170, Solarbio, China). Glutathione can react with 5,5′-dithiobis-2-nitrobenoic acid (DTNB) to produce 2-nitro-5-fluorenyl benzoic acid and glutathione disulfide (GSSG). 2-nitro-5-mercaptobenzoic acid is a yellow product with maximal light absorbance at 412 nm.

NADPH and NADP^+^ were detected through NADPH and NADP^+^ determination kit (Cat# BC1100, Solarbio, China). NADP^+^ and NADPH were extracted from the samples using acidic and basic extracts, respectively. NADPH reduces the oxidized thiazole blue (MTT) to formazan by the hydrogen transfer of phenazine methyl sulfate (PMS), and the absorbance at 570 nm was detected to determine the NADPH content. The NADP^+^ content was determined by reducing NADP^+^ to NADPH using glucose-6-phosphate dehydrogenase.

### Activities of NADPH Oxidase Assays

Ten-day-old seedlings were soaked in 150 mM NaCl solution for 12 h. After treatment, the activities of NADPH oxidase were evaluated according to the method of Wang et al. (2016).

### Western Blot Analysis

Ten-day-old seedlings were soaked in 150 mM NaCl solution for 12 h. After treatment, the protein extraction, SDS-PAGE and subsequent western blot analysis were carried out according to the method of Wang et al. (2008). About 50 μg proteins were solubilized and separated on 12% acrylamide gels (Bio-Rad Mini protein II apparatus). After electrophoresis, the separated proteins were transferred to a polyvinylidene difiuoride membrane, and the membrane was blocked for 90 min with 5% non-fat milk in 0.5% (w/v) Tween 20, 10 mM Tris–HCl (pH 8.0), and 150 mM NaCl. At present, we do not have specific antibodies for different G6PD isoforms. The antibody of G6PD (Sigma) is a polyclonal antibody, which only detects the total protein levels of G6PD. Subsequently, the polyclonal G6PD antibody was added and incubated overnight with the membrane. After washing, alkaline phosphatase-coupled secondary antibody was added and incubated for 2 h. The chemiluminescence was determined with the Pro-light horseradish peroxidase kit (PA112, Tiangen, China). The western blotting images were caught by Tanon-5200 Chemiluminescent Imaging System (Tanon, China).

### Quantitative Real-Time PCR Analysis

Total RNA was extracted with Trizol (TaKaRa) from shoots and roots. RNA was treated with RNase-free DNase (Transgen, China). First-strand cDNA was synthesized with the PrimeScript II 1st Strand cDNA Synthesis Kit (TaKaRa, Transgen, China). Quantitative real-time PCR was performed using the SYBR PrimeScript RT-PCR Kit (Perfect Real Time; TaKaRa). PCR was performed using a CFX 96 Real-Time system (Bio-Rad, Hercules, CA, United States) with the following standard cycling conditions: 95°C for 10 s, followed by 40 cycles of 95°C for 5 s, and 60°C for 30 s. Primer sequences used in the study was shown in Supplementary Table S1. The cycle threshold 2^(-ΔΔC(T))^-based method was used for relative quantitation of gene expression. Expression levels of genes were normalized to *Actin2*.

*Arabidopsis* Genome Initiative locus identifiers for the genes mentioned in this article are as follows: *ACTIN2* (AT3G18780), *G6PD5* (AT3G27300), *G6PD6* (AT5G40760), *G6PD1* (AT5G35790), *G6PD2* (AT5G13110), *G6PD3* (AT1G24280), *G6PD4* (AT1G09420), *AtrbohD* (AT5G47910), *AtrbohF* (AT1G64060), *APX1* (At1G07890), *SOD1* (At1G08830), *POD1* (At1G67960), *CAT1* (At1G20630), *GR2* (At3G54660).

### Generation of the *G6PD5* and *G6PD6* Overexpressing Lines

*Arabidopsis* full-length *G6PD5* or *G6PD6* cDNA was obtained using reverse transcription PCR, cloned into the pENTR-TOPO cloning vector (Invitrogen) and sequenced. After the LR reaction, *G6PD5* or *G6PD6* cDNA was inserted into the pGWB2 vector driven by the 35S promoter; this vector was named pGWB2-*G6PD5* or pGWB2-*G6PD6*. Transformed plants were selected on hygromycin-containing medium. Plants of the second generation after transformation were used for the experiments. The empty pGWB5 vector (the ccdb gene was substituted by a nonsense segment with a termination codon) was also transferred into WT and used as control plants.

### Statistical Analysis

Each experiment was repeated at least three times. Values were expressed as mean ± SE. The data were statistically analyzed using SPSS version 17.0. All comparisons were carried out with one way analysis of variance (ANOVA) followed by Duncan’s multiple range test for independent samples. In all cases, the confidence coefficient was set at *P* < 0.05.

## Results

### Expression Analyses of Cytosolic G6PD

To study the underlying role of cytosolic G6PD in *Arabidopsis*, we obtained T-DNA insertion mutants from the Arabidopsis Biological Resource Center (Supplementary Figure S1A). The results of quantitative real-time PCR and RT-PCR revealed that both *g6pd5* or *g6pd6* are loss-of function null mutants because the *G6PD5* or *G6PD6* transcript level in corresponding mutant was hardly detected (Supplementary Figures S1B,D). In order to further clarify the function of *G6PD5* and *G6PD6*, we generated overexpression lines of *G6PD5* (*OE*#*1* and *OE*#*9*) and *G6PD6* (*OE*#*17* and *OE*#*21*). All overexpression lines showed elevated expression levels of *G6PD5* (4- and 13-fold increase for *OE*#*1* and *OE*#*9*, respectively) or *G6PD6* (6- and 19-fold increase for *OE*#*17* and *OE*#*21*, respectively) (Supplementary Figures S1C,D). Homozygous transgenic plants (*5OE*#*9* and *6OE*#*21*) were chosen for further analysis.

**FIGURE 1:**
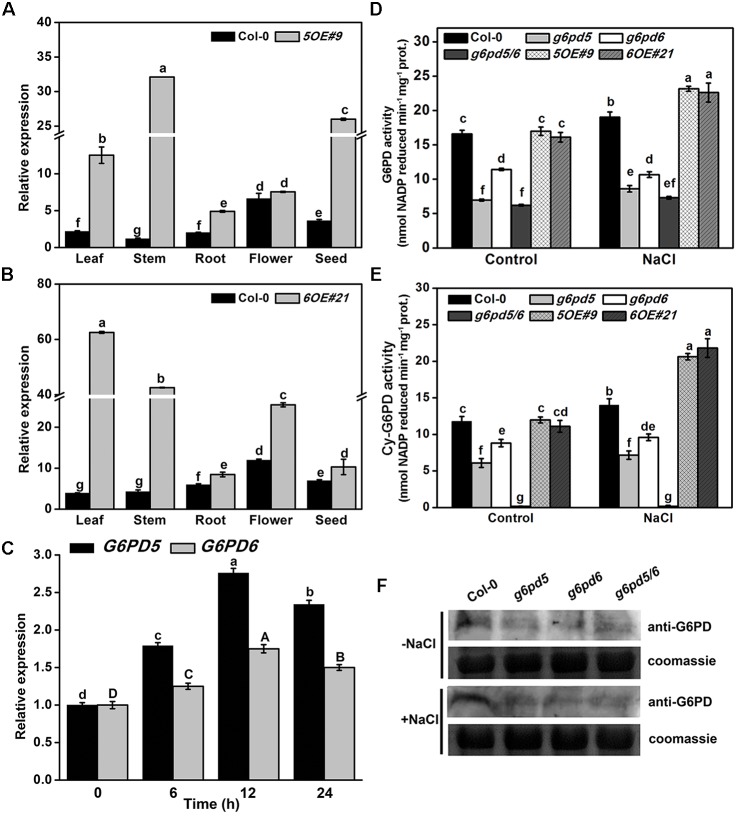
The analysis of cy-G6PD in *Arabidopsis* seedlings with or without salt treatment. **(A,B)** The qRT-PCR analysis of *G6PD5* (WT and *5OE*#*9*) and *G6PD6* (WT and *6OE*#*21*) expression in *Arabidopsis* different organs. **(C)** Relative transcript levels of *G6PD5* and *G6PD6* in wild-type (Col-0) seedlings with the 150 mM NaCl treatment. Uppercase letters represent the error analysis of *G6PD6*, and lowercase letters represent the error analysis of *G6PD5*. **(D,E)** The activities of G6PD or cy-G6PD in *Arabidopsis* WT and mutants exposed to salt treatment. **(F)** Western blot analysis of G6PD expression in *Arabidopsis*. In this experiment, 150 mM NaCl was used for treatment. The Coomassie Brilliant Blue-stained gel was present to show that an equal amount of proteins was loaded in all lanes. Data are mean ± SE of three independent experiments, bars with different letters are significantly different at the level of *P* < 0.05. The experiment was repeated three with similar results.

**FIGURE 2:**
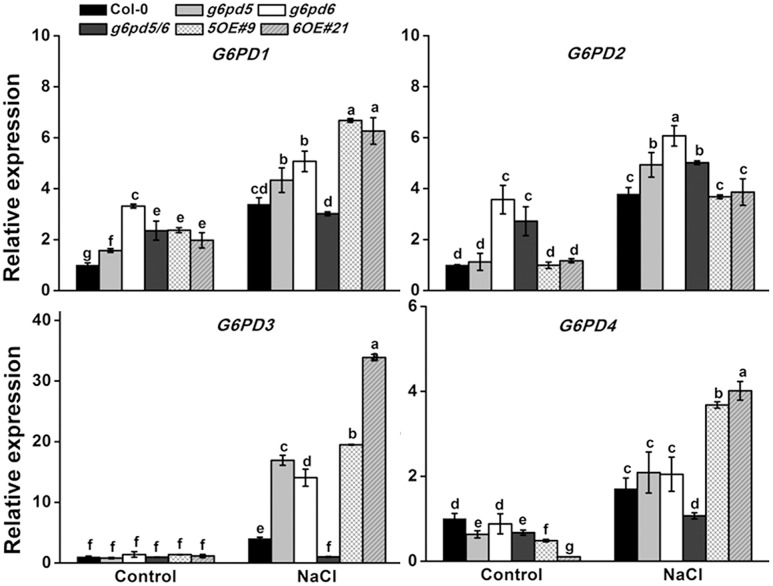
The qRT-PCR analysis of *G6PD*s expression in WT and cy-G6PD mutants. The transcript levels were normalized to *Actin2* gene expression. Results are averages ± SE (*n* = 3), bars with different letters are significantly different at the level of *P* < 0.05. All experiments were repeated at least three times with similar results.

We also found that different *G6PD* family genes have different expression patterns (Supplementary Figure S2 and Figure 1A,B). The high expression level of cytoplasmic *G6PD* (*G6PD5* and *G6PD6*) was observed in all organs examined. The total G6PD enzymatic activities in cy-G6PD loss-of-function mutants were much lower than that in WT seedlings, especially in *g6pd5* and *g6pd5/6* (Figure 1D). Similarly, the activities of cy-G6PD were lower in mutants than in WT (Figure 1E). It was noteworthy that cy-G6PD activity was the main factor in the enhanced total G6PD activity under salt stress, which was responsible for approximately 71% of the total G6PD activity. The western blot results were consistent with the G6PD enzymatic activities in seedlings (Figure 1F). Interestingly, under the normal condition, the expression of *G6PD1* and *G6PD2* in the *g6pd6* or *g6pd5/6* mutants was higher than that in WT. The expression of *G6PD3* in the mutants was similar to WT, whereas the expression of *G6PD4* in the mutants was lower than that in WT with the exception of *g6pd6*. Under salt stress, in single mutants, the expression of *G6PD1*, *G6PD2*, and *G6PD3* was higher than that in WT, while *G6PD4* had no obvious difference compared to WT. In the double mutant, the expression of the *G6PD1* had no obvious difference compared to WT, while *G6PD2* expression was higher than that in WT. The expression of *G6PD3* and *G6PD4* was lower than that in WT (Figure 2).

### Phenotypic Analyses of cy-G6PD Mutants

**FIGURE 3:**
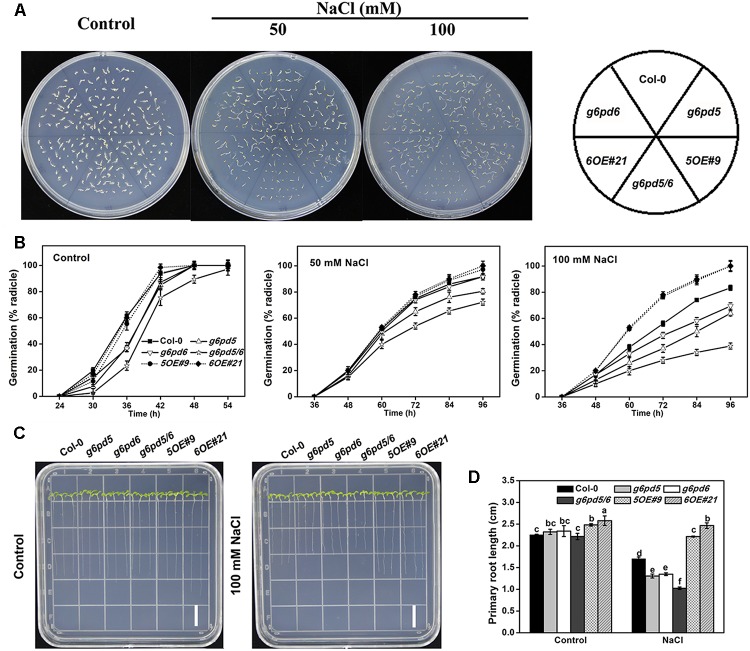
Seed germination and root growth of WT, *g6pd5* mutant, *g6pd6* mutant, *g6pd5/6* mutant, *G6PD5-OE*, and *G6PD6-OE* *Arabidopsis* in response to NaCl stress. Seeds were germinated on ½ MS agar plates with or without various concentrations of NaCl. **(A)** Photographs were taken 3 days in terms of radical emergence after NaCl treatment. **(B)** Percentage of seed germination in WT, *g6pd5* mutant, *g6pd6* mutant, *g6pd5/6* mutant, *G6PD5-OE*, and *G6PD6-OE* with or without different NaCl treatment. **(C,D)** 5-day-old seedlings were grown vertically on ½ MS agar plates supplemented with the indicated concentrations of NaCl for 3 days. Root growth was monitored and analyzed using ImageJ software. Data are reported as the average value of three replicates using >50 seeds for each genotype. One-way Duncan’s test was performed, and statistically significant differences are indicated by different lower case letters (*P* < 0.05). Bar, 1 cm. The experiments were repeated at least three times with similar results, and data from one representative experiment are presented.

Seed germination is a critical phase in the plant life cycle. Successful execution of the germination program greatly depends on the oxidative homeostasis of seeds (Chen et al., 2014b). We evaluated the germination rates of cy-G6PD mutants under different conditions. The germination of the mutant seeds was slightly delayed compared with WT seeds but the mutant seedlings exhibited similar growth rates and plant sizes as WT (Figure 3). To determine the sensitivity of the *g6pd5* or *g6pd6* mutant to salt stress during seed germination and root elongation, different concentrations of NaCl (50 and 100 mM) were supplied in the medium (Figure 3). The results showed that both *g6pd5* and *g6pd6* single mutant exhibited slightly reduced seed germination rate (Figure 3A,B) and primary root length (Figure 3C,D) compared to WT. Because *g6pd5* or *g6pd6* single mutant is not significantly different from WT under salt stress, we generated the double mutant *g6pd5/6* by crossing *g6pd5* with *g6pd6*. RT-PCR results revealed that the transcripts of both *G6PD5* and *G6PD6* in *g6pd5/6* were undetectable (Supplementary Figure S1B). Significantly, the double mutant *g6pd5/6* exhibited low seed germination rate and short primary root length with increased NaCl concentrations compared to WT and single mutants, indicated the function redundancy of *G6PD5* and *G6PD6* (Figure 3). To determine whether the function of cy-G6PD in response to a relative higher concentration of NaCl, we analyzed the seed germination and primary root elongation under 150 mM NaCl treatment. Consistent with those in 50 or 100 mM NaCl treatment, the mutants exhibited more significant salt sensitivity compared with WT (Supplementary Figures S3A,B), whereas the length of primary roots was severely inhibited in 150 mM NaCl (Supplementary Figures S3C,D). NaCl from 50 to 150 mM promoted obvious cy-G6PD accumulation, but only 50 or 100 mM NaCl had significant effects on *Arabidopsis* stress tolerance.

Under normal growth conditions, slight difference in the germination and primary root growth was observed between the WT and the overexpression lines (Figure 3). However, under salt stress, the OE lines exhibited a significantly higher seed germination rate than WT (Figure 3A,B) and the root growth of OE plants was less sensitive to NaCl treatment (Figure 3C,D). These data indicate that overexpression of cy-G6PD increases salinity tolerance in *Arabidopsis*.

We also examined the transcript level of *G6PD5* and *G6PD6* in *Arabidopsis* seedlings under salt treatment using qRT-PCR. In accordance with our previous results, the expression of *G6PD5* and *G6PD6* in WT seedlings was significantly induced by salt stress (Figure 1C). In summary, *G6PD5* and *G6PD6* are involved in seed germination and root growth under salinity in *Arabidopsis.*

### Response to Oxidative Damage in cy-G6PD-Overexpressing and cy-*g6pd* Mutant Plants

**FIGURE 4:**
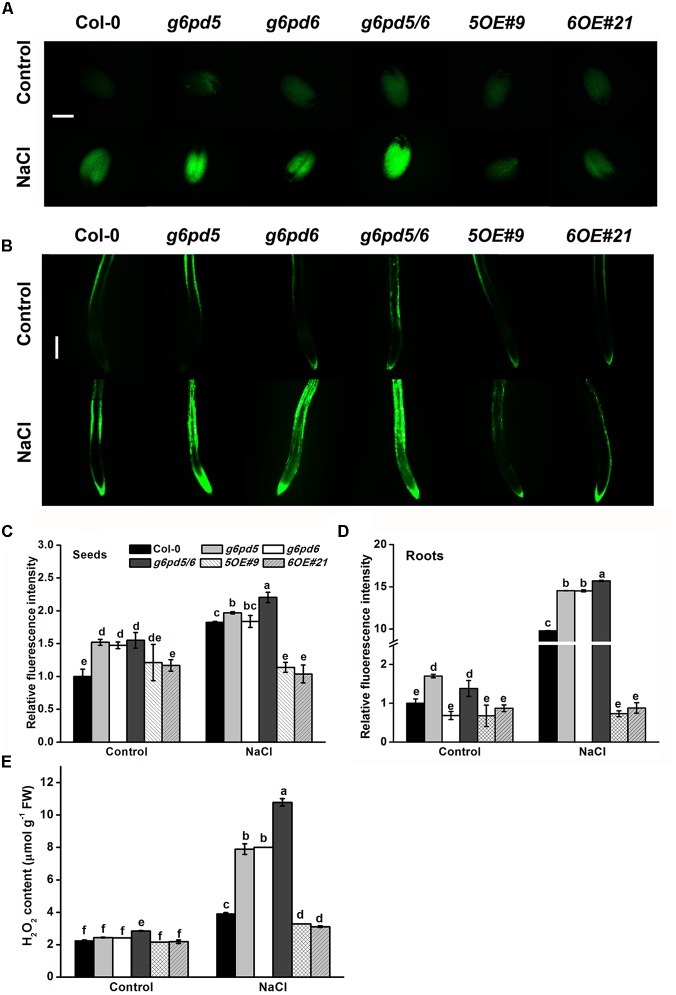
The *g6pd5*, *g6pd6*, and *g6pd5/6* mutant affect the ROS levels under salt stress. 1-day-old seeds and 5-day-old seedlings were grown vertically on ½ MS agar plates supplemented with the 150 mM NaCl for 12 h. **(A)** The levels of H_2_O_2_ were measured using the H_2_DCF-DA fluorochrome dyes in *Arabidopsis* seeds. Bar, 200 μm. **(B)** The levels of H_2_O_2_ were measured using the H_2_DCF-DA fluorochrome dyes in *Arabidopsis* roots. Bar, 200 μm. **(C,D)** Quantification of the fluorescence in *Arabidopsis* seeds and roots under NaCl treatment. **(E)** 10-day-old seedlings were grown vertically on ½ MS agar plates supplemented with the 150 mM NaCl for 12 h. Data are mean ± SE of three independent experiments, bars with different letters are significantly different at the level of *P* < 0.05. The experiment was repeated three with similar results.

Reactive oxygen species (ROS) play a key regulatory role in the germination program under salt stress (Chen et al., 2014a). The ROS levels in cy-*g6pd* mutants and WT under NaCl stress were explored in this study. As shown in Figure 4, the ROS content in seeds and roots of both cy-*g6pd* mutant and WT was increased in response to NaCl treatment. It is noteworthy that such effects were significantly enhanced in the *g6pd5/6* double mutant but attenuated in OE lines (Figure 4). Additionally, ROS content analysis revealed significantly higher levels of H_2_O_2_ in the double mutant than WT in seedlings under salt treatment, which was consistent with previous findings in seeds and roots (Figure 4E). To further dissect the role of cy-G6PD involvement in ROS signaling, exogenous H_2_O_2_ was supplied to the medium. The double mutant *g6pd5/6* showed increased sensitivity to the oxidative stress, as manifested by delayed germination and retarded root elongation relative to WT (Supplementary Figures S4A,B). In contrast, OE lines exhibited reduced sensitivity to the oxidative stress (Supplementary Figures S4A,B). Moreover, exogenous application of diphenyliodonium iodide (DPI), an inhibitor for H_2_O_2_, partially rescued the root growth phenotype of *g6pd5/6* (Supplementary Figure S4C). These results suggest that the oxidative level is higher in *g6pd5/6* than in WT.

### cy-G6PD Influences the Expression of NADPH Oxidases AtrbohD and AtrbohF

**FIGURE 5:**
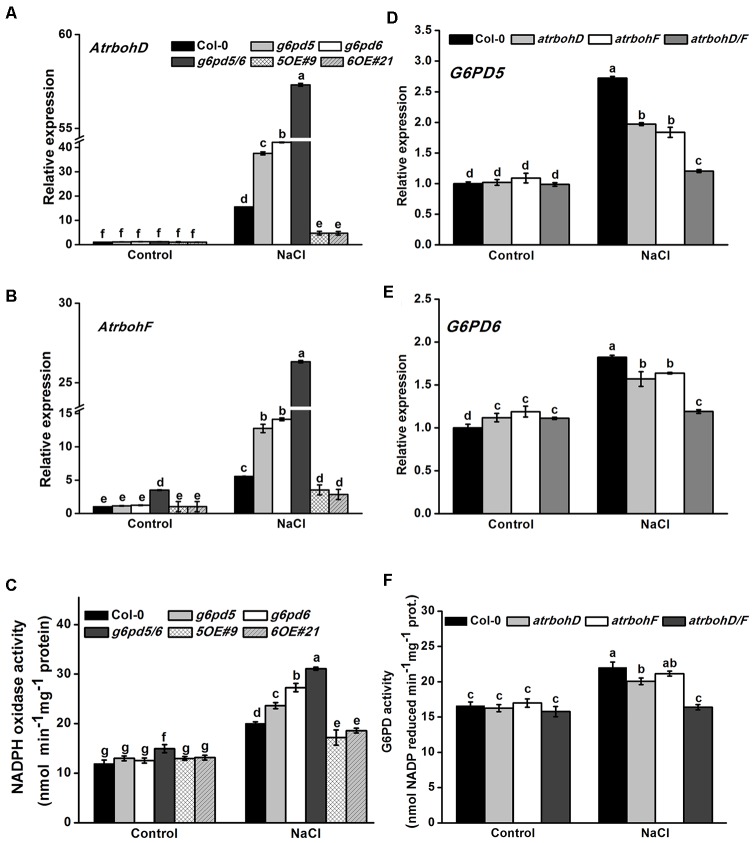
The response of G6PD5 and G6PD6 to salt stress through NADPH oxidases signaling pathway. **(A,B)** Relative transcript levels of NADPH oxidases AtrbohD and AtrbohF genes in *Arabidopsis* seedlings with or without 150 mM NaCl treatment. **(C)** The activities of NADPH oxidase in *Arabidopsis* WT and mutants exposed to salt treatment. **(D,E)** Relative transcript levels of *G6PD5* and *G6PD6* in WT and NADPH oxidases mutant seeds (*atrbohD1*, *atrbohF1*, and *atrbohD1/F1*) exposed to salt treatment. The transcript levels were normalized to *Actin2* gene expression. **(F)** The activities of G6PD in *Arabidopsis* WT and mutants exposed to salt treatment. Results are averages ± SE (*n* = 3), bars with different letters are significantly different at the level of *P* < 0.05. All experiments were repeated at least three times with similar results.

Plasma membrane NADPH oxidase is considered to be an important producer of ROS, which has been shown to play a role in plant acclimation to salt stress (Ma et al., 2012; Jiao et al., 2013). In addition, the NADPH oxidases AtrbohD and AtrbohF are important in stress-inhibited primary root growth in *Arabidopsis* (Ma et al., 2012). To determine whether the function of cy-G6PD in response to salt stress is achieved through the NADPH oxidase signaling pathway, we analyzed the expression of NADPH oxidases genes in WT, *g6pd5*, *g6pd6*, and OE plants with or without salt treatment. As shown in Figure 5, the expression of *AtrbohD* and *AtrbohF* was markedly increased by salt treatment in all materials, and the salt-induced gene expression levels in *g6pd5/6* was significantly higher than that in WT plants (Figure 5A,B). Consistently, the activity of NADPH oxidase was also higher in *g6pd5/6* than in WT under salt stress (Figure 5C). These results suggest that cy-G6PD is involved in RBOH-dependent ROS production in salt-stressed seedlings. To prove the hypothesis, we examined the expression of *G6PD5* and *G6PD6* in NADPH oxidase mutants, *atrbohD1* (CS9555), *atrbohF1* (CS9557), and *atrbohD1/F1* (CS9558). In these mutants, the expression of both *G6PD5* and *G6PD6* was lower than that in WT plants (Figure 5D,E). As expected, the G6PD enzymatic activity in *atrboh* loss-of-function mutants was also lower than that in WT, especially in *atrbohD1/F1* (Figure 5F).

### cy-G6PD Affects the Intracellular NADPH Levels Under Salt Stress

**FIGURE 6:**
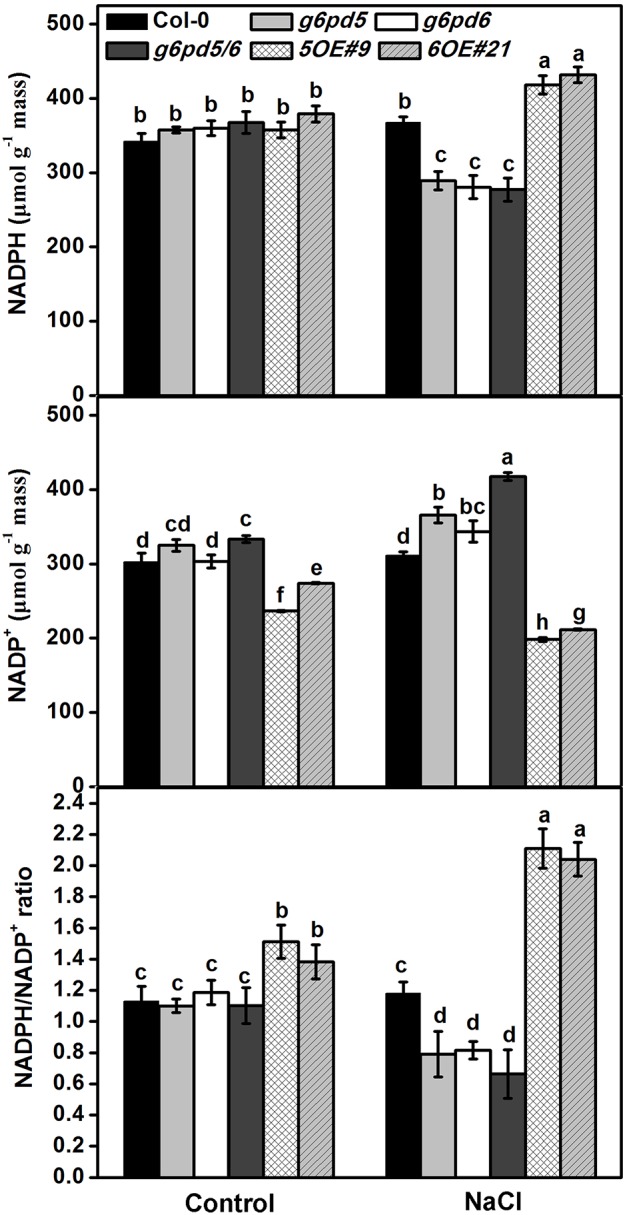
G6PD5 and G6PD6 affects NADPH content in *Arabidopsis* under salt stress. 10-day-old seedlings were grown vertically on ½ MS agar plates supplemented with the 150 mM NaCl for 12 h. Results are averages ± SE (*n* = 3), bars with different letters are significantly different at the level of *P* < 0.05. All experiments were repeated at least three times with similar results.

As a reducing power, NADPH is the substrate of the NADPH oxidase. NADPH oxidase uses NADPH to generate superoxide, which can be dismutated subsequently to hydrogen peroxide (Wang et al., 2008; Stampfl et al., 2016; Wang et al., 2016). Moreover, the NADPH/NADP^+^ ratio is considered a possible mechanism for G6PD regulation (Cardi et al., 2011, 2015). Thus, NADPH is a key connector between G6PD and the ROS scavenging system. Figure 6 showed that cy-G6PD affected the intracellular NADPH and NADP^+^ levels and the NADPH/NADP^+^ ratio. Consistent with the reduced cy-G6PD activity, the intracellular NADPH level and the NADPH/NADP^+^ ratio were significantly decreased in *g6pd5/6* mutant plants exposed to salt stress. In salt-stressed *cy-G6PD*-overexpression plants, the NADPH level and the NADPH/NADP^+^ ratio were higher than that in WT (Figure 6), indicating that G6PD is important for the intracellular NADPH homeostasis.

### cy-G6PD Enhances the Expression of Antioxidant Responsive Genes

Antioxidant enzymes are responsive to stresses to scavenge extra ROS to maintain the balance between ROS production and scavenging (Liu et al., 2015). To investigate the effects of *cy-G6PD* on the expression of antioxidant enzymes, we determined the activities and expression levels of antioxidant enzymes, including *APX, CAT, GR, POD*, and *SOD* (Supplementary Figures S5A,B). The results showed that the salt stress-induced activity and expression levels of *APX* and *GR* in the *g6pd5/6* mutant were significantly lower than that in WT plants (Supplementary Figures S5A,B). In contrast, the expression levels of *APX* and *GR* in OE lines were higher than that in WT (Supplementary Figures S5A,B). These results suggest that cy-G6PD involvement in the regulation of seed germination and root growth is mediated by APX and GR and that cy-G6PD enhances the capacity of plants to scavenge excessive ROS under salt stress to maintain the balance between ROS production and scavenging. The *g6pd5/6* mutant is more sensitive to oxidative damages caused by salt stress because it has reduced ROS scavenging capability. These data indicate that enhanced cy-G6PD activity provides more NADPH for the antioxidant system to remove excess ROS.

### cy-G6PD Enhances Glutathione Levels Under Salt Stress

Glutathione (GSH), one of the essential antioxidants and redox buffers, is involved in plant development as well as tolerance to various stresses (Wang et al., 2008). As shown in Supplementary Figure S6, the GSH content was increased by salt treatment in WT seedlings. Consistent with the role of cy-G6PD in redox regulation, the GSH level was decreased in *g6pd5/6* under salt stress (Supplementary Figure S6). These results indicated that G6PD is essential for the glutathione level.

Our further analysis showed that exogenous application of ascorbate acid (ASC) or glutathione partially or fully rescued the seed germination and root growth phenotype in *g6pd* single and double mutants (Supplementary Figure S7). It was noteworthy that GSH was more effective than ASC (Supplementary Figure S7). In short, cy-G6PD participates in the reduction of H_2_O_2_ to H_2_O possibly through the glutathione peroxidase cycle or the ascorbate-glutathione cycle.

### cy-G6PD Is Required for Cell Elongation and Root Meristem Maintenance

**FIGURE 7:**
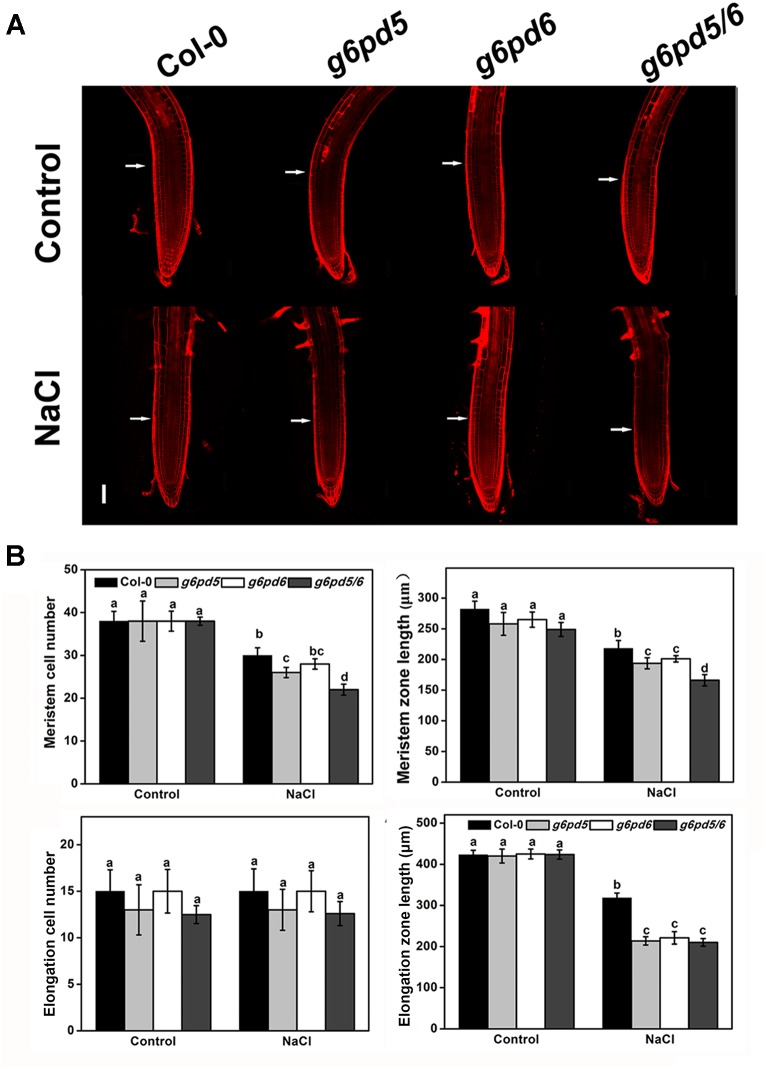
G6PD5 and G6PD6 regulate root meristem and elongation zone. **(A)** Root meristems of propidium iodide (PI)-stained images in *Arabidopsis* WT seedlings. The meristem zone was marked with white arrows in **(A)**. Bars = 100 μm. **(B)** Root meristem cell number, meristem zone size, elongation cell number, and elogation zone size in *Arabidopsis* WT seedlings. The 5-day-old seedlings were treated with 100 mM NaCl for 12 h. Mean values and SE were calculated from three independent experiments (*n* = 20). Within each set of experiments, bars with different letters were significantly different at the 0.05 level.

Previous studies have shown that ROS can control root elongation by loosening cell walls and inhibiting cell division (Liszkay et al., 2004; Jiao et al., 2013). To further dissect the mechanisms of cy-G6PD function in salt-repressed root growth in *Arabidopsis*, we measured the primary roots length in WT and *cy-G6PD* mutants supplied with 100 mM NaCl. The root meristem length was evaluated by determining the number of cortical cells in the region from the quiescent center (QC) to the first-elongated cell (Dello Ioio et al., 2007). The root growth of WT and mutants was similar on NaCl-free medium (Figure 7). However, *g6pd* mutant plants had shortened root elongation zone compared to WT after growing on NaCl-containing medium for 12 h (Figure 7A,B). These results indicate that NaCl suppresses the enlargement of the elongation zone in roots of *cy-G6PD* mutants relative to WT. In addition to cell elongation in the elongation zone, cell division in the root meristem zone also contributes to root growth. Therefore, we also determined the size of root apical meristem. The number of meristem cells in *g6pd* mutant plants was less than that in WT in the presence of NaCl, implying that cy-G6PD is required for cell division in the root meristem (Figure 7A,B).

## Discussion

G6PDs have critical functions in plant development and stress responses (Wang et al., 2008, 2016). cy-G6PD plays a key role in plant adaptation to various stresses in several species (Dal Santo et al., 2012; Stampfl et al., 2016; Wang et al., 2016). The aim of this study was to elucidate the function and regulatory mechanism of cy-G6PD in *Arabidopsis* response to salt stress. The high expression level of *cy-G6PD* in various organs of *A. thaliana* suggests its important function (Supplementary Figure S2). Under normal condition, the expression of the *G6PD1* and *G6PD2* (both are plastidial *G6PD*s) in the *g6pd5/6* mutants is higher than that in WT (Figure 2), suggesting that plastidial *G6PD*s may have function redundancy with *cy-G6PD*s, but this notion still needs to be further proved.

In this study, the involvement of cy-G6PD in the response of to salt stress was investigated during seed germination and root development in *Arabidopsis* (Figure 3). Our results showed that *G6PD5* and *G6PD6* play central roles in seed germination and seedling growth under unfavorable conditions. The seed germination rate of the double mutant *g6pd5/6* was reduced by approximately 60% compared to WT under salt stress, implying that cy-G6PD is involved in this process (Figure 3). It was reported that the G6PD activity is increased in drought-stressed soybean seedlings and the drought-tolerant cultivar shows higher G6PD activity than the drought-sensitive cultivar (Liu et al., 2013; Wang et al., 2016). However, how stress activates the cy-G6PD activity and the regulatory roles of cy-G6PD in stress tolerance need further clarification. Thus, we characterized the *g6pd5/6* mutant, which is hypersensitive to salt stress during seed germination and root elongation of seedlings. With the genetic evidence, we further determined the function of *cy-G6PD* in response to stress conditions. Overexpression of *G6PD5* or *G6PD6* results in enhanced tolerance, whereas the *g6pd5/6* mutant shows attenuated tolerance compared to WT.

In addition, we demonstrated that cy-G6PD inhibited the ROS generation in the germination program under salt stress (Figure 4). ROS are the ever-present danger due to their physicochemical toxicity that they accumulate under many stress conditions (Liu et al., 2015; Wang et al., 2016). Previous reports showed that H_2_O_2_ increases the G6PD activity in red kidney bean roots and reed callus under salt stress (Wang et al., 2008; Liu et al., 2012). Furthermore, H_2_O_2_ plays a role in drought-induced increase of the total G6PD activity (Wang et al., 2016). Our results of H_2_O_2_ on *G6PD5* and *G6PD6* suggest that cy-G6PD is enhanced to scavenge the excessive ROS under salt stress in order to maintain the balance between ROS production and scavenging, and that the enhanced cy-G6PD activity provides more NADPH for the antioxidant system to remove excessive ROS (Figure 4).

Glutathione peroxidase cycle and ascorbate-glutathione cycle can catalyze the reduction of H_2_O_2_ to water. We investigated the role of cy-G6PD in regulating the levels of reduced form of glutathione (GSH) under salt stress and found that G6PD is involved in GSH maintenance and H_2_O_2_ accumulation (Supplementary Figures S5, S6). In plants, NADPH could be generated by ferredoxin-NADP reductase and four NADP-dehydrogenases: G6PD, 6-phosphogluconate dehydrogenase (6PGD), NADP-isocitrate dehydrogenase, and the NADP-malic enzyme (Leterrier et al., 2016). Loss-of-function of cy-G6PD dramatically decreases the intracellular NADPH level and the NADPH/NADP^+^ ratio under salt stress (Figure 6), suggesting that G6PD contributes to the major part of NADPH production in *Arabidopsis* seedlings. A similar decrease in the GSH content was observed (Supplementary Figure S6). Overexpression of *cy-G6PD* leads to the enhanced GSH pool and oxidative tolerance by providing more NADPH. From the above results, we concluded that cy-G6PD, the major contributor to the total G6PD activity, is the key factor for maintaining intracellular GSH and NADPH levels under salt stress; disruption of the NADPH and GSH homeostasis resulted in oxidative damages in *Arabidopsis* seedlings.

Understanding the roles of G6PD and NADPH oxidases will increase our knowledge of the plant ROS network in developmental and physiological challenges. As key ROS-generating enzymes, NADPH oxidases AtrbohD and AtrbohF are essential components for numerous biological processes (Chaouch et al., 2012; Jiang et al., 2013). The expression and activities of the NADPH oxidases are markedly increased by salt treatment (Figure 5). This is consistent with previous findings that an NADPH oxidase inhibitor (DPI) interfered with a defense-induced ROS burst after salt stress, and that the *Arabidopsis* double mutant *rbohD/F* exhibits decreased cy-G6PD enzymatic activities. These results suggest that cy-G6PD is involved in RBOH-dependent ROS production in salt-stressed seedlings.

In plants exposed to high salinity, G6PD contributes to ROS detoxification and the maintenance of cellular redox balance (Dal Santo et al., 2012). However, in addition to their damaging role in plants challenged by prolonged salt stress, ROS also have important signaling functions. RBOHD is involved in regulating ROS signaling in response to salinity (Miller et al., 2009, 2010; Baxter et al., 2014). cy-G6PD might also be involved in RBOH-dependent ROS production and signaling in salt-stressed plants (Stampfl et al., 2016). In this study, the expression of NADPH oxidase genes *AtrbohD* and *AtrbohF* in salt-induced *g6pd5/6* seedlings is higher than that in control plants, however, expression levels of *APX* and *GR* in the *g6pd5/6* mutant is significantly lower than that in WT plants. These results suggest that the high levels of ROS in *g6pd5/6* plants may be sufficient to activate antioxidative defense systems. Undoubtedly, the dual role of cy-G6PD in ROS scavenging and generation in *Arabidopsis* still needs to be further illustrated.

## Conclusion

**FIGURE 8:**
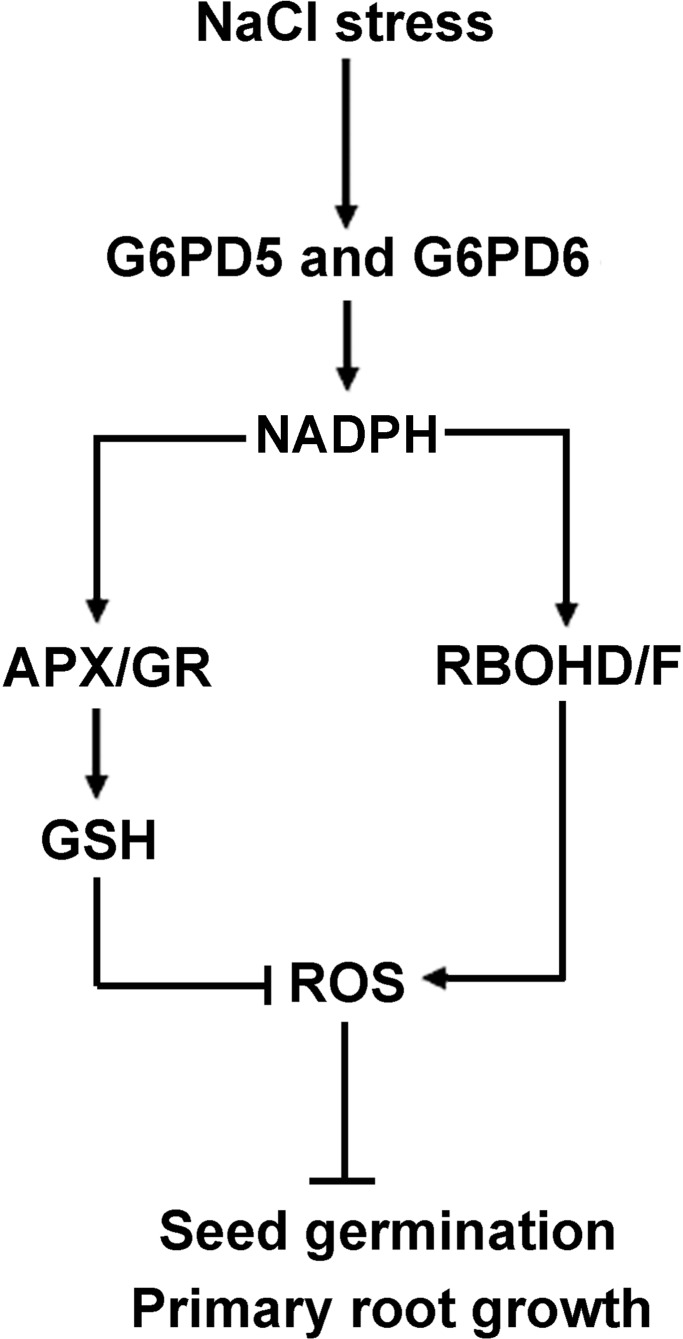
Schematic illustration of a proposed model for the link between G6PD5, G6PD6, ROS, and APX-GR in *Arabidopsis* seed germination and root growth. In this model, arrows indicate positive regulation, bars indicate negative regulation. Salt stress induces cy-G6PD, which subsequently maintain the intracellular NADPH homeostasis, and involved in regulating key enzymes (APX and GR) in ASC-GSH cycle. The APX and GR inhibit the level of H_2_O_2_ in cells through GSH content. cy-G6PD is involved in H_2_O_2_ accumulation through applying NADPH to PM NADPH oxidase. The enhanced cy-G6PD thus control germination of *Arabidopsis* seeds and growth of *Arabidopsis* primary roots.

Our results showed that H_2_O_2_, NADPH, RBOHD/F, APX/GR, and GSH are required for salt-induced *cy-G6PD* gene function, and that the enhanced cy-G6PD plays an important role against oxidative stress by increasing the ASC and GSH levels, which in turn dampen ROS accumulation. Our findings point to a different node of this crosstalk that is activated by an increase in the cytosolic H_2_O_2_ and that is involved in dormancy, germination control, and the stress responsiveness of seeds. Based on the results presented here, we proposed a hypothetical model shown in Figure 8. In this model, salt stress induces *cy-G6PD*. The enhanced *cy-G6PD* is involved in regulating key enzymes (APX and GR) in ASC-GSH cycle by utilizing NADPH, which eventually results in the increased ASC and GSH levels. The enhanced antioxidant ability can maintain a steady-state level of H_2_O_2_ in cells, thus avoiding ROS damages. cy-G6PD is also involved in RBOH-dependent ROS production in salt-stressed seedlings. Moreover, cy-G6PD is involved in root apical meristem (RAM) maintenance through the glutathione redox-affected ROS pathway.

## Author Contributions

LY, YB, XW, and WN conceived and designed the experiments. LY, NC, SW, MR, LS, and SL performed the experiments. LY analyzed the data. LY and YB wrote the manuscript. All authors reviewed the manuscript.

## Conflict of Interest Statement

The authors declare that the research was conducted in the absence of any commercial or financial relationships that could be construed as a potential conflict of interest.

## Footnotes

1 http://www.arabidopsis.org/
