# Supplementary material for: Cytosolic Glucose-6-Phosphate Dehydrogenase Is Involved in Seed Germination and Root Growth Under Salinity in Arabidopsis
Source: Front Plant Sci. 2019 Feb 22;10:182. doi: 10.3389/fpls.2019.00182 (PMC6401624; doi:10.3389/fpls.2019.00182)
Supplement: Supplementary file 1 [file Data_Sheet_1.pdf]

Cytosolic glucose-6-phosphate dehydrogenase is involved in seed germination and root growth under salinity in *Arabidopsis*

Lei Yang[1], Xiaomin Wang[1,2], Ning Chang[1], Wenbin Nan[3], Shengwang Wang[1], Mengjiao Ruan[1], Lili Sun[1], Sufang Li[1] and Yurong Bi[1*]

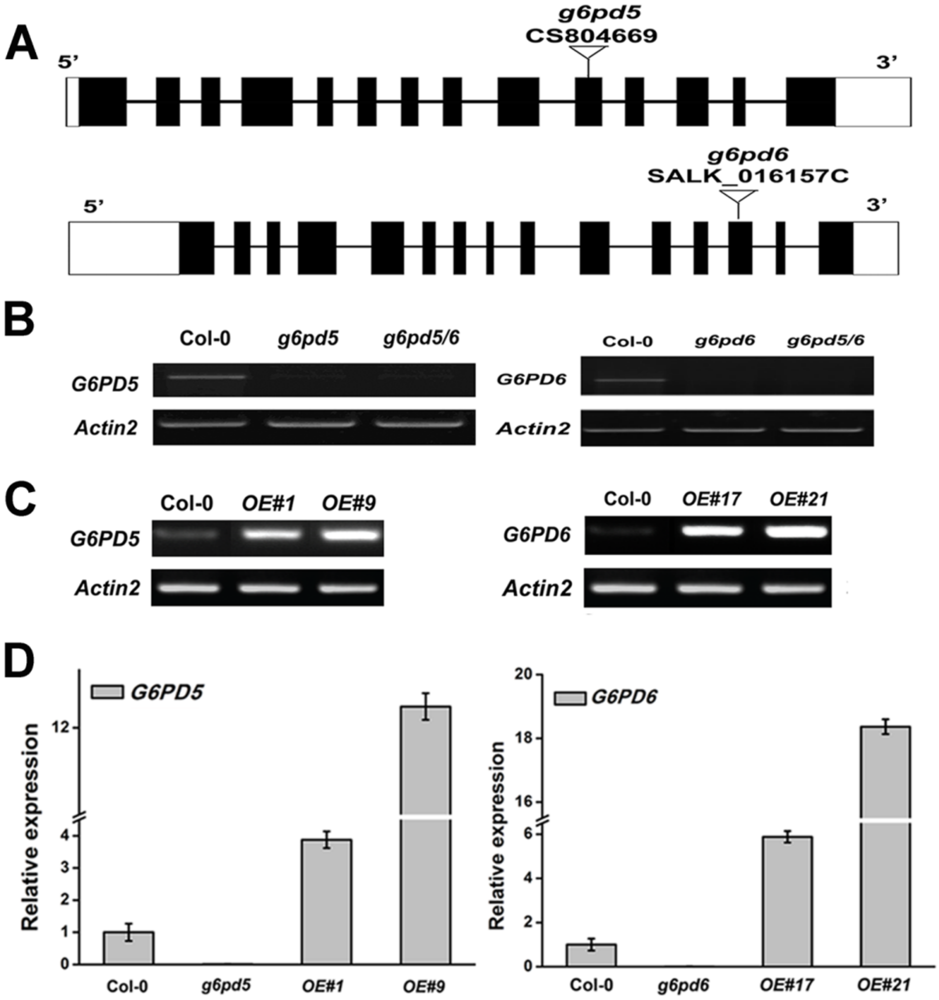

FIGURE S1| The gene structure and expression of *G6PD5* and *G6PD6* in *Arabidopsis* seedlings. (A) The diagram of T-DNA insertion position in *g6pd5* and *g6pd6* mutants. Exons are represented by boxes (untranslated regions in white and coding sequence in black) and introns by the black line. The T-DNA insertions into the gene are shown as triangles. (B) RT-PCR analysis of the relative transcript levels of *G6PD5* and *G6PD6* in *g6pd5*, *g6pd6*, or *g6pd5/6* seedlings; *Actin2* was used to standardize gene expression. (C) Real-time RT-PCR analysis for *G6PD5* and *G6PD6* transcript in overexpression plants. (D) Real-time RT-PCR analysis for *G6PD5* transcript in total RNA from the Col-0, *g6pd5*, *OE#1* and *OE#9* plants and *G6PD6*

transcript in total RNA from the Col-0, *g6pd6*, *OE#17* and *OE#21* plants. The transcript levels all were normalized to *Actin2* gene expression. The transcript levels were normalized to *Actin2* gene expression. Results are averages ± SE (n = 3). All experiments were repeated at least three times with similar results.

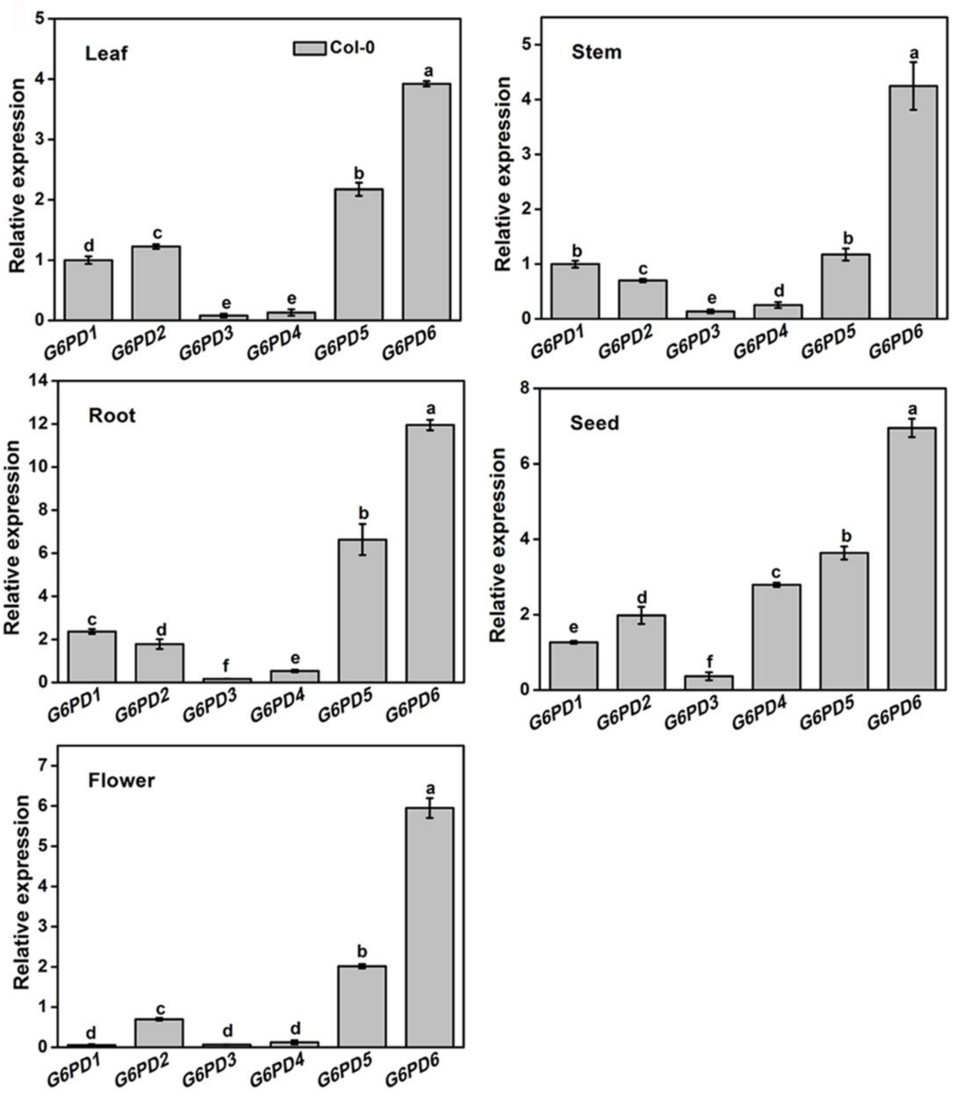

FIGURE S2| The qRT-PCR analysis of *G6PD* expression in *Arabidopsis* different organs. The transcript levels were normalized to *Actin2* gene expression. Results are averages ±SE (n = 3), bars with different letters were significantly different at the 0.05 level. All experiments were repeated at least three times with similar results.

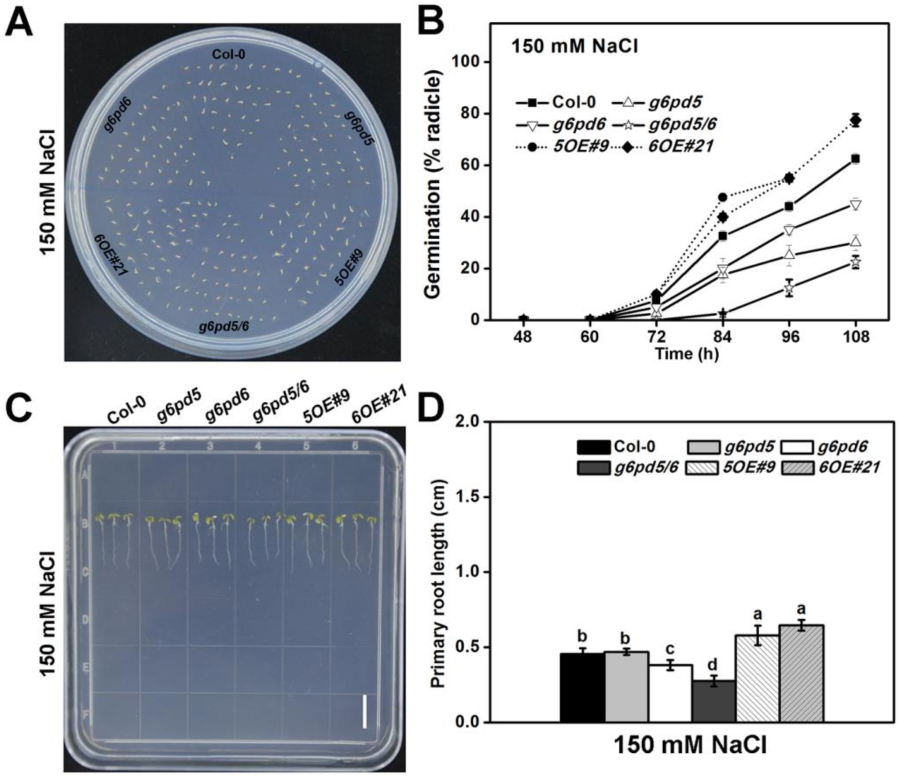

FIGURE S3| Seed germination and root growth of WT, *g6pd5* mutant, *g6pd6* mutant, *g6pd5/6* mutant, *G6PD5-OE,* and *G6PD6-OE Arabidopsis* in response to NaCl stress. (A) Photographs were taken 5 days in terms of radical emergence after NaCl treatment. (B) Percentage of seed germination in WT, *g6pd5* mutant, *g6pd6* mutant, *g6pd5/6* mutant, *G6PD5-OE*, and *G6PD6-OE* with 150 mM NaCl treatment. (C) and (D) 5-day-old seedlings were grown vertically on ½ MS agar plates supplemented with the indicated concentrations of NaCl for 3 days. Root growth was monitored and analyzed using ImageJ software. Data are reported as the average value of three replicates using>50 seeds for each genotype. One-way Duncan's test was performed, and statistically significant differences are indicated by different lower case letters ($P<0.05$). Bar, 1cm. The experiments were repeated at least three times with similar results, and data from one representative experiment are presented.

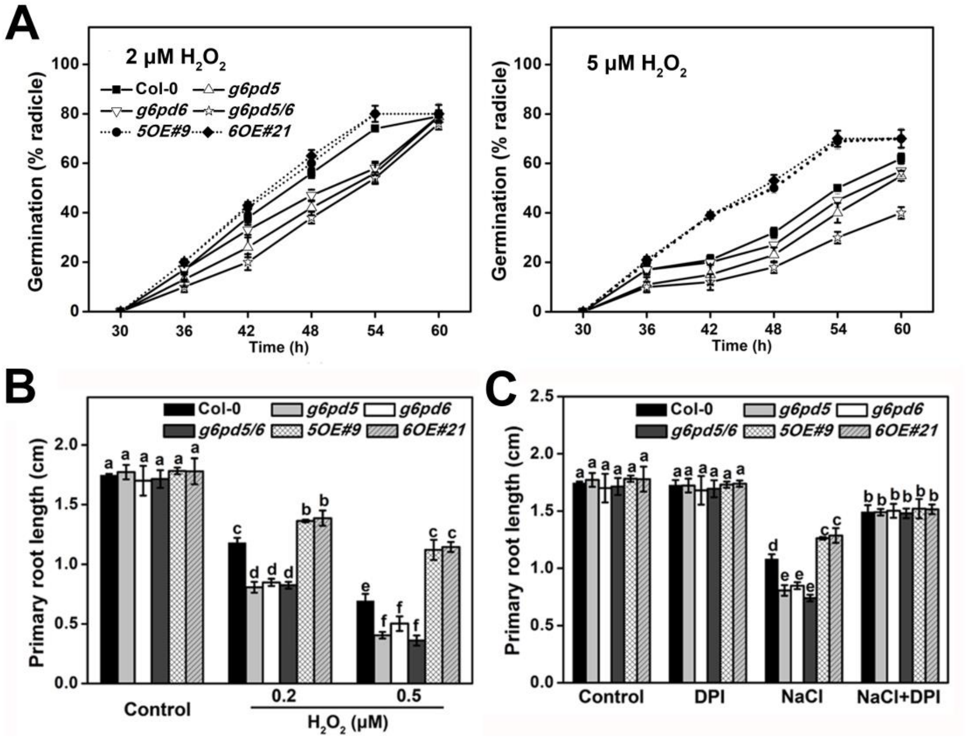

FIGURE S4| The *g6pd5*, *g6pd6* and *g6pd5/6* mutant response to oxidative stress under adding exogenous H_2O_2. (A) Germination assay was conducted on 1/2 MS agar plates containing different concentrations of H_2O_2. (B) Root growth of WT, *g6pd5*, *g6pd6*, *g6pd5/6* mutant, *G6PD5-OE*, and *G6PD6-OE Arabidopsis* plants in response to H_2O_2. (C) Effect of exogenous DPI (2 μM) on root growth of *g6pd5*, *g6pd6* and *g6pd5/6* mutant. Data are presented as mean values ± SD of three independent experiments. One-way Duncan's test was performed, and statistically significant differences are indicated by different lower case letters ($P<0.05$). Bar, 1cm. The experiments were repeated at least three times with similar results, and data from one representative experiment are presented.

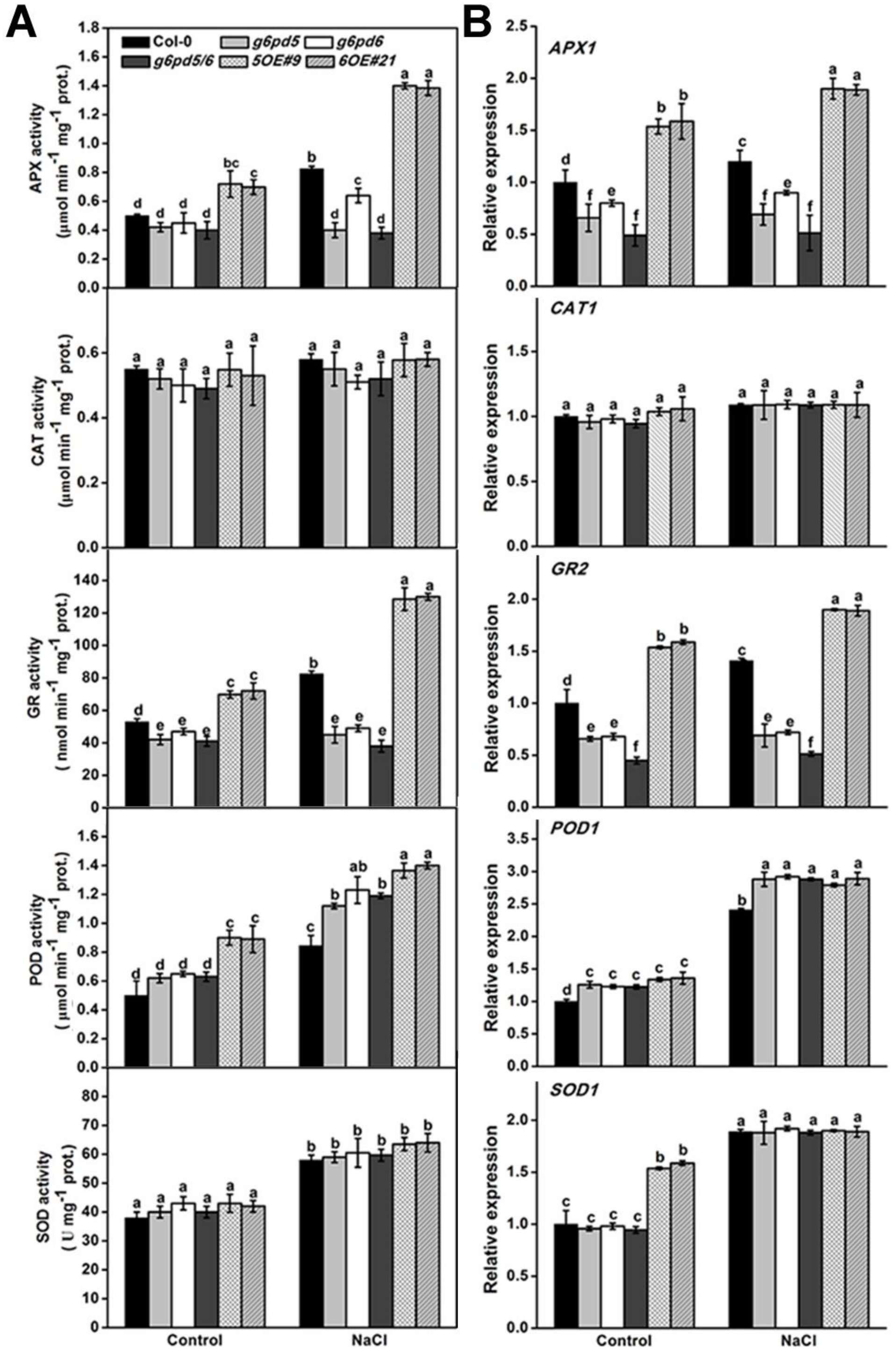

FIGURE S5| Effects of G6PD5 and G6PD6 on the activities of antioxidant enzymes and transcript levels of antioxidant enzymes responsive genes in *Arabidopsis* seedlings exposed to salt treatment. (A) and (B) The transcript levels were normalized to *Actin2* gene expression. Results are averages ±SE (n = 3), bars with different letters were significantly different at the 0.05 level. All experiments were repeated at least three times with similar results.

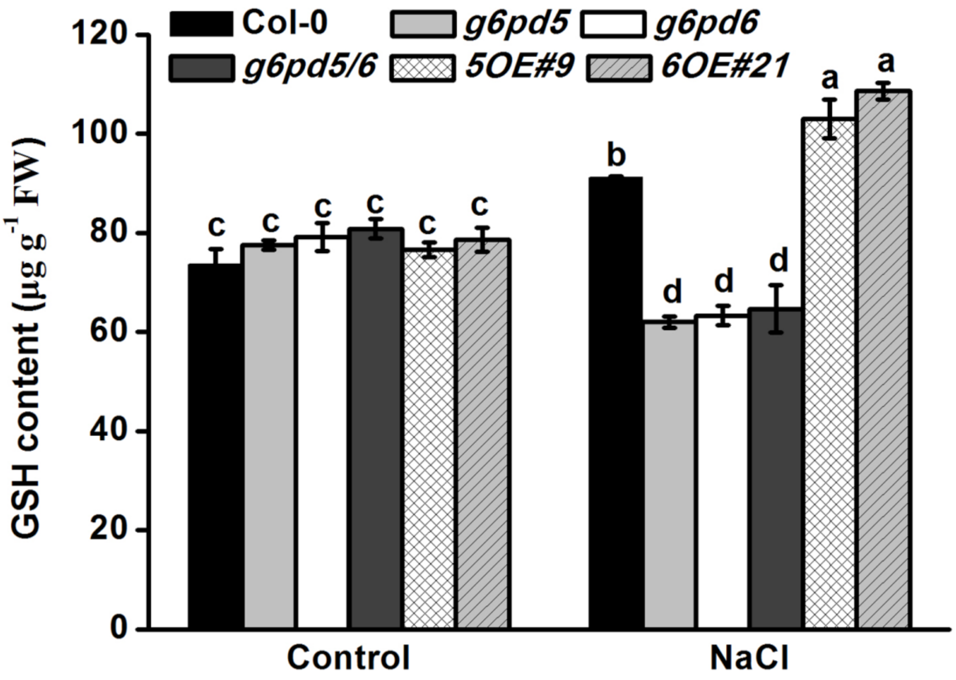

FIGURE S6| Change of GSH contents in WT, *g6pd5* mutant, *g6pd6* mutant, *g6pd5/6* mutant, *G6PD5-OE*, and *G6PD6-OE Arabidopsis* under salt stress. The seedlings were treated as in Supplemental Figure 5. Mean values and SE were calculated from three independent experiments. Within each set of experiments, bars with different letters were significantly different at the 0.05 level.

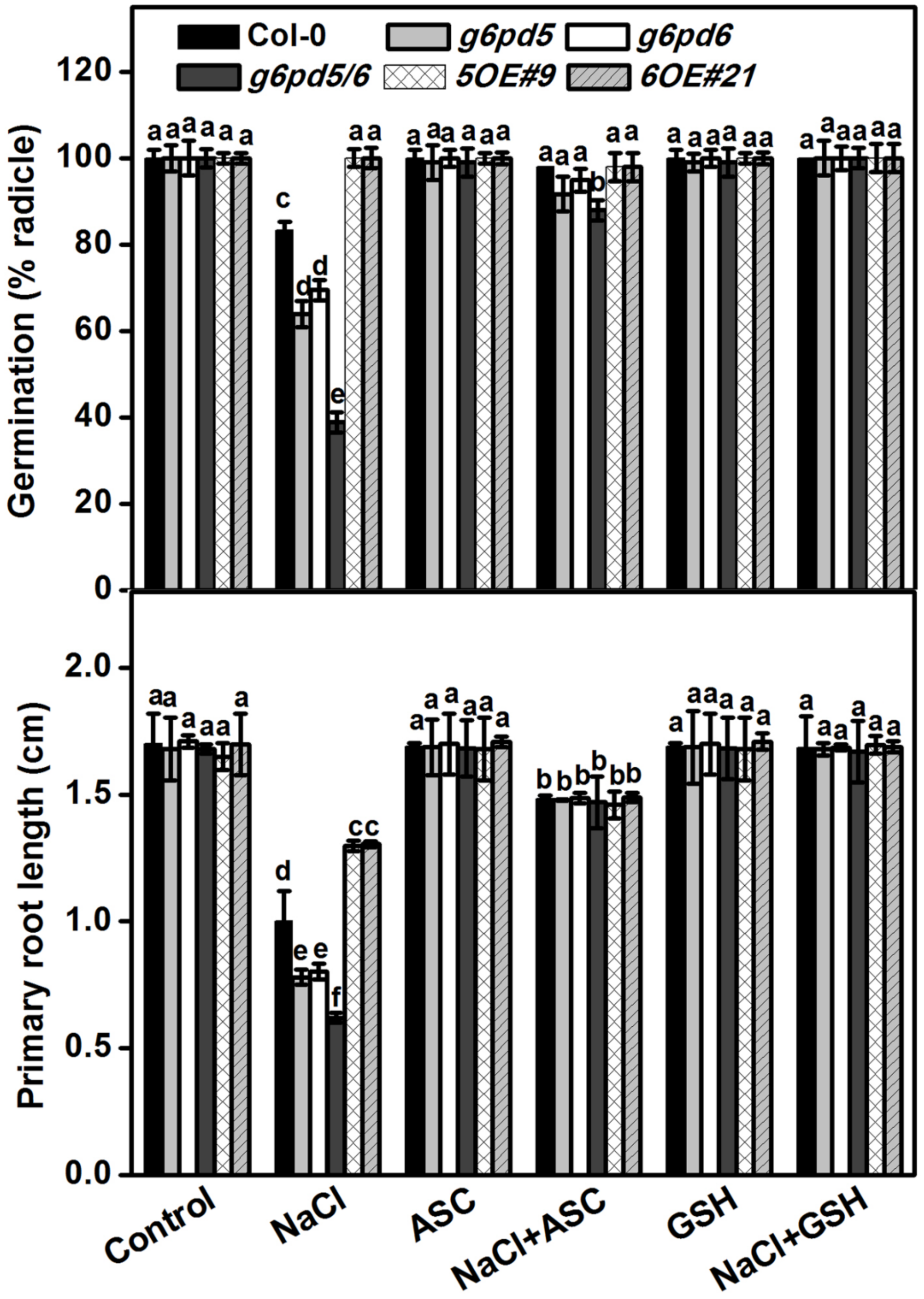

FIGURE S7| Effect of exogenous ASC and GSH on seed germination and root growth of *g6pd5*, *g6pd6* and *g6pd5/6* mutant. The seeds and seedlings are incubated with 0.25 μM ASC solution or 5 μM GSH. Data are presented as mean values ± SD of three independent experiments. One-way Duncan's test was performed, and statistically significant differences are indicated by different lower case letters ($P<0.05$). The experiments were repeated at least three times with similar results, and data from one representative experiment are presented.

Table S1 Primer sequences used in the study

Gene name	Primer sequence 5’-3’
Actin2	GTT GGG ATG AAC CAG AAG GA<br>CTT ACA ATT TCC CGC TCT GC
qActin2	TTTCCCGCTCTGCTGTTGT<br>TGTGCCAATCTACGAGGGTTT
G6PD5	CACCATGGGTTCTGGTCAATGGC<br>CAATGTAGGAGGGATCTAAATGTAG
qG6PD5	TCTTGCACTTCCTCCGTCTG<br>GCGTTCGTAAGCCTCTGG
G6PD6	CACCATGGGATCTGGTCAATGG<br>TAGTGTAGGAGGGATCCAGATATAGC
qG6PD6	GTTGGTCCTCCGGTTTGC<br>CTTTCGGTCCTCGGCTTC
qG6PD1	GGTCAATACAAAGGCCATAA<br>AACAGGACCTCTGCTTCC
qG6PD2	TCAATGGATGGACTAGGGTTA<br>TGGACACGGATCACAAGC
qG6PD3	CGCTATGGTCAAGGCAGTA<br>CATCGCTTCTTATGAACAATCT
qG6PD4	TTACCTATCAGTACCTCAAGAAGCT<br>ATATTTTCTCGGTATAGGTTTCCAG
qAtrbohD	TGGAAGGATGGACTGGCATT<br>CTTGAGGAAGTTAGGTAAGTTAAGC
qAtrbohF	GACTTCTCAGAGCCGACGAA<br>CAATGCCAAGACCAACTAATAAGAG
qAPX1	GTGTTTTTGGTTGGGGGCTG<br>GTCTAAGCAGCAAAAGCGCA
qSOD1	CCAGGAAGGCGATGGTGTGA<br>CCAGTAGACATGCAACCGTTAGTG
qPOD1	ATGACTTACTACATGATGAGCTGTCC<br>CAGTGTTGTCTTTCGTTGAATCTAG

qCAT1	GTGGAATCTCTTCGTTCAGGTGATG<br>GTTCAAGACCAAGCGACCAACAG
qGR2	GGTCGCAAGCCCAACACAAAG<br>ACAGCCCAGATGGATGGAACAG
LBb1	GCGTGGACCGCTTGCTGCAACT
pGWB2	ATTTGGAGACACGGGG
